# HIV Epidemiology in Africa: Weak Variables and Tendentiousness Generate Wobbly Conclusions

**DOI:** 10.1371/journal.pmed.0020137

**Published:** 2005-05-31

**Authors:** Stuart Brody, John J Potterat

**Affiliations:** **1**University of PaisleyPaisleyScotland; **2**Colorado Springs, Colorado, United States of America

In their attempt to defend heterosexual transmission as the driving force for HIV epidemics in sub-Saharan Africa and dismiss the evidence for unsafe injections (or other percutaneous exposures) as a major source of HIV transmission [1,2], Lopman and colleagues [3] assert that “unsafe medical injections can be confidently excluded as a major source of HIV infection” in Manicaland, Zimbabwe. Their methods and results are glaringly insufficient to generate the confidence they seek. Confidence requires thorough evaluation, which was not done in their study [3].

Although the authors quantified the number of sexual partners, they failed to quantify the number of injections [3], which undermines the likelihood of detecting an effect of injections, as does adding the statistical noise of “needle pricks” (how was that question phrased?) to injections. We find it difficult to believe that the authors were unaware of either the dose-dependency issue or the implications of using a weak measure of a vector that they are transparently invested in dismissing. In addition, the recall interval was inordinately long (up to three years). It is unreasonable to expect accurate data from subjects who are not multiply prompted to stimulate recall.

As a secondary issue, their adjustment of the risk ratio associating HIV with injections against age is problematic. Adjusting a causal variable against age (which is a proxy for causal variables) may reduce the true association. Their Table 1 shows that injections in women vary by age in the same pattern that HIV incidence varies by age. It might be that when they adjust for age, the risk ratios for age capture the association, leaving a diminished association for injections.

Confidence in their report is also undermined by their approach to their own finding that sexual behavior was unrelated to risk of incident HIV. Indeed, using the same standard of evidence that the authors applied to their null medical-injection finding, the abstract, discussion, and press release information should also have proclaimed that sex was “confidently excluded” as a risk for HIV. Indeed, this would be consistent with the largest studies of HIV risk in Africa, including one in their own backyard (Manicaland) [4], which found little association of measured sexual behaviors with HIV risk. It would also be consistent with the many intervention studies reporting no benefit from condom-promotion programs [5], as well as with observation of the opposite trajectories of the HIV and STD epidemics observed in Zimbabwe [6].

Of additional concern is Lopman et al.'s finding that 13 of 67 individuals who seroconverted reported no sexual partners in the long inter-survey period. Indeed, their Table 2 data show that women with no reported sexual partners have higher HIV incidence than women who report any partner during the three-year interval (1.56 HIV cases per 100 person-years [12/770] for the former compared to 1.21 [36/2975] for the latter). They reveal their a priori conviction by forcing this datum into the procrustean sexual bed, inferring that it is explainable by unreported sexual activity rather than unreported or unmeasured percutaneous exposure. The authors blame underreporting of sexual behavior without using techniques shown to dramatically improve valid reporting [7].

Despite this remarkable lack of association in their women respondents between HIV incidence and number of sexual partners, they counterintuitively suggest (in the patient summary) that the important issue is sexual transmission. It seems to us that if sex doesn't appear to explain high HIV incidence, then one should recommend looking for what does, which the authors do not do. Rather than relying on the case-control approach, what is needed is intensive contact tracing (with viral sequencing of HIV specimens from index cases and their infected contacts to elucidate transmission relationships [8]) and a rigorous inventory of possible exposures and vectors [7]. The contact tracing and conscientious environmental risk probing of recent public-health responses to avian influenza and severe acute respiratory syndrome are an example of a useful and superior step to the case-control approach.

In brief, what is truly driving HIV transmission in sub-Saharan Africa will not be resolved by hastily implemented weak variables or by dismissive comments about Africans' self-reports of sexual behavior, especially comments unencumbered by data. Africans, science, and public health deserve better research [7].
